# Cross-cultural adaptation of the Young Disability Questionnaire-Spine into Turkish for children with spinal pain: a methodological validation study

**DOI:** 10.1136/bmjopen-2026-120763

**Published:** 2026-10-09

**Authors:** Abdullah İyigün, Caner Günerbüyük, Pervin Demir, Ömer Polat, Kürşat Güner, Mehmet Atıf Aksekili

**Affiliations:** 1 Department of Orthopaedics and Traumatology, Umraniye Training and Research Hospital, Istanbul, Turkey; 2 Department of Orthopaedics and Traumatology, Koç University Faculty of Medicine, Istanbul, Turkey; 3 Department of Biostatistics, Yildirim Beyazit University Faculty of Medicine, Ankara, Turkey; 4 Department of Orthopaedics and Traumatology, Medicana International Ankara Hospital, Ankara, Turkey

**Keywords:** Back pain, Child, Surveys and Questionnaires, Cross-Sectional Studies, STATISTICS & RESEARCH METHODS

## Abstract

**Abstract:**

**Objectives:**

To translate and culturally adapt the Young Disability Questionnaire-Spine (YDQ-spine) into Turkish and evaluate the structural validity, reliability and construct validity of the Turkish version (T-YDQ-spine) in children aged 9–12 years with spinal pain.

**Design:**

Methodological validation study.

**Setting:**

A tertiary paediatric orthopaedics centre in Istanbul, Türkiye.

**Participants:**

A total of 199 children aged 9–12 years with spinal pain were included. The first 59 completed the T-YDQ-spine again after 5 days and 99 also completed the Turkish Scoliosis Research Society-22 (SRS-22).

**Primary and secondary outcome measures:**

Structural validity was assessed using an ordinal correlated two-factor confirmatory factor analysis with weighted least squares mean-adjusted and variance-adjusted estimation. Internal consistency was evaluated using Cronbach’s alpha, ordinal alpha and McDonald’s omega; test–retest reliability using absolute-agreement intraclass correlation coefficients (ICCs); and construct validity using eight predefined hypotheses based on Spearman correlations with SRS-22 domains. Floor and ceiling effects were also examined.

**Results:**

The correlated two-factor model showed very good fit (Comparative Fit Index=0.997, Tucker-Lewis Index=0.996, root mean square error of approximation=0.018, standardised root mean square residual=0.045), with a latent factor correlation of 0.586. Cronbach’s alpha was 0.929 for the physical and 0.901 for the psychosocial component; corresponding McDonald’s omega values were 0.941 and 0.916. Test–retest ICCs were 0.918 and 0.898, respectively. Seven of eight predefined construct-validity hypotheses (87.5%) were confirmed. No floor or ceiling effects were observed for either component; the standalone sleep item showed a floor effect.

**Conclusions:**

The T-YDQ-spine demonstrated evidence supporting structural validity, internal consistency, test–retest reliability and construct validity in Turkish-speaking children aged 9–12 years with spinal pain. Further multicentre studies should evaluate responsiveness, longitudinal interpretability and cross-cultural measurement invariance.

**STRENGTHS AND LIMITATIONS OF THIS STUDY**

- The Turkish adaptation of the Young Disability Questionnaire (YDQ)-spine followed a structured cross-cultural adaptation process, including forward and backward translation, expert review and pretesting in children from the target age group.
- Structural validity was evaluated using an ordinal correlated two-factor confirmatory factor analysis, and reliability assessment included multiple internal-consistency indices, absolute-agreement test–retest reliability and measurement-error estimates.
- Construct validity was evaluated using predefined hypotheses against conceptually related and less closely related domains of the Turkish Scoliosis Research Society-22 (SRS-22) rather than relying solely on the magnitude of correlation coefficients.
- Participants were recruited from a single tertiary paediatric orthopaedics centre, and detailed pain characteristics such as localisation, intensity and duration beyond the minimum eligibility criterion were not systematically collected, limiting subgroup analyses and generalisability.
- The SRS-22 is not an age-specific criterion measure for non-specific spinal pain, and responsiveness and cross-cultural measurement invariance between the Turkish and original Danish versions were not assessed.

## Introduction

 Spinal pain is increasingly recognised in children and adolescents and may be associated with limitations in daily activities, school absenteeism, reduced participation in social and physical activities and impaired health-related quality of life.^1–3^ Its occurrence during childhood is multifactorial and may reflect a combination of biological, behavioural, psychosocial and environmental influences.^4^ Importantly, spinal pain experienced at a young age may persist or recur over time, underscoring the need for appropriate assessment and follow-up during childhood.^5^

Patient-reported outcome measures (PROM) are important for evaluating how spinal pain affects everyday functioning from the child’s own perspective. However, many disability instruments used for spinal pain were originally developed for adults and may not adequately reflect the developmental level, language, daily activities and psychosocial context of younger children.^6^ The Young Disability Questionnaire-Spine (YDQ-spine) was specifically developed for children aged 9–12 years with spinal pain and was derived from domains identified as important by children themselves.^6 7^ It assesses the consequences of spinal pain across physical and psychosocial domains, includes a separate sleep item, and provides an optional section addressing the aspects of spinal pain that matter most to the child.^8^ This age-specific and child-centred development process distinguishes the YDQ-spine from instruments that were not originally designed for younger children.

The YDQ-spine was developed and field-tested in Danish schoolchildren, with the final instrument comprising 24 items organised into a 13-item physical component, a 10-item psychosocial component and 1 separately scored sleep item.^8^ Although its original development established the conceptual framework and factor structure, further evaluation of its measurement properties in other languages and cultural settings is required before it can be used confidently across populations. No validated Turkish version is currently available for assessing spinal pain-related disability specifically in children aged 9–12 years. A culturally adapted Turkish version may therefore address an important gap in age-specific assessment and facilitate the use of a standardised child-reported measure in both clinical practice and research in Türkiye.

The aim of this study was to translate and culturally adapt the YDQ-spine into Turkish and to evaluate its structural validity, internal consistency, test–retest reliability, construct validity and floor and ceiling effects in Turkish-speaking children aged 9–12 years with spinal pain.

## Methods

### Study design and participants

This methodological validation study was conducted at a tertiary paediatric orthopaedics centre in Istanbul, Türkiye. Participants were recruited consecutively between October 2024 and October 2025. Eligibility criteria were age 9–12 years, spinal pain lasting at least 1 week, ability to read and understand Turkish and sufficient cognitive ability to complete the questionnaires independently. Children with neurological or developmental disorders, congenital spinal deformities, other musculoskeletal disorders affecting questionnaire completion, cognitive impairment or insufficient command of Turkish were excluded. Written informed consent for research participation was obtained from the parents or legal guardians of all participants.

A total of 199 children were included in the main psychometric sample. This sample was used for the confirmatory factor analysis of the 23 ordinal items representing the physical and psychosocial components; the single Sleep item was analysed separately. Pain localisation, pain intensity and detailed pain duration beyond the minimum 1-week eligibility criterion were not systematically collected and were therefore unavailable for subgroup analyses.

Sample sizes for structural validity, test–retest reliability and construct-validity analyses were determined before recruitment. For the confirmatory factor analysis, the planned main sample was informed by simulation evidence showing that adequacy depends on model complexity, number of indicators and factor-loading magnitude rather than a fixed participant-to-item ratio.^9^ For test–retest reliability, 59 participants were planned based on published sample-size guidance for questionnaire reliability studies using intraclass correlation coefficients.^10^ For the correlation analyses, 99 participants were planned to provide acceptable precision for correlations of the expected magnitude.^11^

Test–retest reliability was evaluated in the first 59 participants included in the study, who completed the Turkish version-YDQ (T-YDQ)-spine again 5 days after the initial assessment. The 5-day interval was selected to minimise recall of previous responses while limiting the likelihood of substantial clinical change between assessments. Construct validity against the Turkish Scoliosis Research Society-22 (SRS-22) was assessed in a subsample of 99 participants who completed both instruments.

### Measurement tools

#### Young Disability Questionnaire-Spine

The YDQ-spine was developed to assess the consequences of spinal pain in children aged 9–12 years within the framework of the International Classification of Functioning, Disability and Health.^6–8^ It is a child-reported instrument comprising two sections.

Section 1 contains 24 items. Of these, 13 items form the physical component and 10 items form the psychosocial component, while 1 sleep item is scored separately. Each Section 1 item is rated on a five-category ordinal scale from 0 (never) to 4 (most of the time), with higher scores indicating greater spinal pain-related disability. Physical and psychosocial component scores are calculated separately and transformed to a 0–100 scale according to the developer’s scoring instructions. A component score is calculated only when no more than three physical items or two psychosocial items are missing; otherwise, the corresponding component score is considered missing. A total score combining the physical and psychosocial components is not recommended and was not calculated in this study. The sleep item is retained as a separate 0–4 score.

Section 2 is optional and consists of four impact items addressing pain, limitations in physical activities, limitations in social relations and the effect of pain on mood and concentration. Each item is rated independently on a 0–10 scale and is not included in the Section 1 component scores. In the present study, these items were analysed separately to describe the relative importance attributed to each impact area and to assess their test–retest reliability.

### Scoliosis Research Society-22

The Turkish-validated version of the SRS-22 questionnaire was used as an external comparator for construct validity.^12^ The SRS-22 assesses health-related quality of life across domains including pain, function/activity, self-image, mental health and satisfaction with management, with higher scores indicating better health status. Although the SRS-22 was originally developed primarily for individuals with scoliosis and spinal deformity, it was selected because several of its domains conceptually overlap with the physical and psychosocial consequences assessed by the YDQ-spine. Its use therefore allowed predefined hypotheses to be tested regarding the expected direction and magnitude of associations between related and less closely-related constructs. The limitations associated with using the SRS-22 outside its primary clinical context are addressed in the Discussion.

### Translation and cross-cultural adaptation

The Turkish adaptation of the YDQ-spine followed the cross-cultural adaptation framework proposed by Beaton *et al*.^13^ Permission to translate and culturally adapt the questionnaire into Turkish was obtained from the instrument developer, Henrik Hein Lauridsen, by email on 18 December 2023. The English YDQ-spine version available at that time was used as the source instrument.

According to the revision history provided by the developer, the English YDQ-spine was updated on 23 February 2024. The update affected only the question stem and response anchors of the four optional Section 2 impact items; the 24-item Section 1 structure, item content and scoring were unchanged. Because the Turkish adaptation process had already commenced using the English version current on 18 December 2023, the study used that source version. The final Turkish version, including its wording, instructions, response options and layout, together with the translation documentation, was subsequently submitted to the developer and received formal approval. The T-YDQ-spine questionnaire is provided as online supplemental material 1.

The adaptation process comprised five stages:

1. Forward translation: Two bilingual translators independently translated the English questionnaire into Turkish.
2. Synthesis: The two forward translations were reviewed and reconciled into a single Turkish version.
3. Backward translation: Two different bilingual translators, blinded to the original questionnaire, independently back-translated the synthesised Turkish version into English.
4. Expert committee review: A multidisciplinary committee comprising an orthopaedic specialist, physiotherapist, language expert and paediatric psychologist reviewed the translations for semantic, idiomatic and conceptual equivalence.
5. Pretesting: The pre-final Turkish version was administered to 15 children from the target age group to assess comprehensibility and cultural appropriateness. Two expressions were revised following pretesting before enrolment of the main study sample. In item 17, the initial translation of ‘get upset’ as ‘kederlenirim’ was changed to ‘hüzünlenirim’ because the latter was more readily understood by the children. In item 24, ‘chiropractor’, initially translated as ‘kayropraktör’, was replaced with ‘masör’ because several children were unfamiliar with the former term. This was the wording administered to the main study sample. During peer review, the use of ‘masör’ was reconsidered because it is not professionally or semantically equivalent to ‘chiropractor’. Rather than reintroducing ‘kayropraktör’, which had not been understood by several children during pretesting, ‘masör’ was removed from the questionnaire in the final publication version, leaving ‘doctor’ and ‘physiotherapist’. The item stem, response options and scoring were unchanged.

### Statistical analysis

Statistical analyses were performed using R V.4.4.2.^14^ Continuous variables were summarised as mean±SD or median (IQR), as appropriate, and categorical variables as frequencies and percentages. Item-level missingness and response distributions were examined for all Section 1 and Section 2 items.

YDQ-spine component scores were recalculated from item-level responses according to the developer’s scoring instructions and cross-checked against stored scores. Physical and psychosocial scores were considered missing when more than three or two component items, respectively, were missing. No overall YDQ-spine total score was calculated.

Structural validity of Section 1 was assessed using confirmatory factor analysis (CFA) with lavaan.^15^ All 23 physical and psychosocial items were entered simultaneously into the prespecified correlated two-factor model (13 physical; 10 psychosocial); the standalone sleep item was excluded. Ordinal items were estimated with weighted least squares mean-adjusted and variance-adjusted estimation using polychoric correlations, with pairwise present observations for missing data.^16^ Model fit was evaluated using the robust χ² statistic and df, χ²/df, Comparative Fit Index (CFI), Tucker-Lewis Index (TLI), root mean square error of approximation (RMSEA) with 90% CI and close-fit test and standardised root mean square residual (SRMR). Standardised factor loadings and the latent factor correlation were estimated. Separate one-factor models were retained only as supplementary sensitivity analyses.

Internal consistency was evaluated separately for the physical and psychosocial components using psych,^17^ with Cronbach’s alpha (bootstrap 95% CI), ordinal alpha and McDonald’s omega total. Corrected item-total correlations and alpha if item deleted were also examined. Internal consistency was not calculated for the single sleep item.

Test–retest reliability was evaluated in the first 59 participants, who completed the T-YDQ-spine again after 5 days, using the irr package.^18^ Reliability of the physical and psychosocial component scores was quantified using the intraclass correlation coefficient for absolute agreement and a single measurement, intraclass correlation coefficient (ICC)(A,1) in the McGraw and Wong nomenclature,^19^ equivalent to ICC(2,1) in the Shrout and Fleiss classification,^20^ selected and reported according to published guidance.^21^ The same two-way, absolute-agreement, single-measurement ICC model was applied to each of the four Section 2 impact items. ICCs were reported with 95% CIs. The SE of measurement (SEM) was calculated as $SD_{baseline}\sqrt{1-ICC}$, using the baseline SD in the test–retest subsample and the 95% minimal detectable change (MDC_95_) was calculated as $1.96\times \sqrt{2}\times SEM$.^22^ Bland-Altman mean differences and 95% limits of agreement and Wilcoxon signed-rank tests were examined as supplementary assessments of systematic change between administrations. For the ordinal sleep item, quadratic-weighted kappa with a 95% bootstrap CI was used as the primary agreement measure^23^; linear-weighted kappa and exact percentage agreement were examined as sensitivity measures, and kappa values were interpreted using established benchmarks.^24^

Construct validity was evaluated using hypothesis testing against the Turkish SRS-22 in the 99 participants who completed both instruments, in accordance with established recommendations for the evaluation of PROMs.^25 26^ Because higher YDQ-spine scores indicate greater disability whereas higher SRS-22 scores indicate better quality of life, negative associations were expected. Eight hypotheses were specified regarding the direction, magnitude and relative strength of correlations between YDQ-spine scores and SRS-22 domains. Spearman’s rank correlation coefficients (ρ) with bootstrap 95% CIs and two-sided p values were calculated. The five individual SRS-22 domains—pain, self-image, function/activity, mental health and satisfaction with management—were used for formal hypothesis testing; composite subtotal and total scores were reported descriptively but were not counted as separate hypotheses. Construct validity was considered supported when at least 75% of the predefined hypotheses were confirmed.^25^

Floor and ceiling effects were evaluated as at least 15% of participants achieving the minimum or maximum possible score for the physical component, psychosocial component or sleep item.^25^

The four optional Section 2 impact items were analysed individually using median, IQR, observed range, missingness and ICC(A,1); no Section 2 summary score was calculated.

As an exploratory analysis, measurement invariance of the correlated two-factor model across sex was examined for ordered categorical indicators using sequential configural, threshold, metric and scalar models, following recommendations for ordinal CFA.^27^ Changes in model fit were interpreted using predefined thresholds of ΔCFI ≥−0.010 and ΔRMSEA ≤0.015.^28^ This analysis assessed invariance across sex only and was not considered evidence of cross-cultural measurement invariance between the Turkish and original versions. Results of these exploratory analyses are reported in online supplemental material 2, table S5.

All statistical tests were two-sided, with p<0.05 considered statistically significant where hypothesis testing was applicable. Statistical decisions based on predefined psychometric thresholds were made using unrounded estimates.

### Patient and public involvement

Patients and the public were not involved in the design, conduct, reporting or dissemination plans of this research.

## Results

### Participant characteristics and data quality

A total of 199 children were included (mean age 10.65±1.08 years); 87 (43.7%) were girls and 112 (56.3%) were boys (table 1).

**Table 1:** Participant characteristics and analysis samples

Characteristic	Value
Total sample, n	199
Age, years, mean±SD	10.65±1.08
Age, years, median (range)	11 (9–12)
Female, n (%)	87 (43.7)
Male, n (%)	112 (56.3)
Test–retest subsample, n	59
SRS-22 construct-validity subsample, n	99

Data are presented as n (%), mean±SD or median (range), as appropriate. The first 59 participants completed the T-YDQ-spine again after 5 days for test–retest assessment. Construct validity against the SRS-22 was evaluated in 99 participants.

SRS-22, Scoliosis Research Society-22 .

Section 1 and Section 2 data were complete for all participants. No duplicate identifiers or score-recalculation discrepancies were identified. The test–retest and SRS-22 construct-validity subsamples comprised 59 and 99 participants, respectively (table 1).

### Structural validity

The prespecified correlated two-factor CFA showed very good fit (robust χ²(229)=243.37, p=0.245; CFI=0.997; TLI=0.996; RMSEA=0.018, 90% CI 0.000 to 0.035; SRMR=0.045), with a latent physical-psychosocial correlation of 0.586 (SE=0.052, p<0.001) (table 2).

**Table 2:** Structural validity, internal consistency and test–retest reliability of the T-YDQ-spine

Psychometric property	Physical component	Psychosocial component	Overall/sleep
Structural validity: correlated two-factor CFA, n=199			
Robust χ² (df), p	—	—	243.37 (229), 0.245
CFI	—	—	0.997
TLI	—	—	0.996
RMSEA (90% CI)	—	—	0.018 (0.000 to 0.035)
SRMR	—	—	0.045
Latent factor correlation	—	—	0.586
Internal consistency, n=199			
Number of items	13	10	Sleep: 1
Cronbach’s α (95% CI)	0.929 (0.912 to 0.942)	0.901 (0.876 to 0.920)	Not applicable
Ordinal α	0.940	0.916	Not applicable
McDonald’s ω	0.941	0.916	Not applicable
Test–retest reliability, n=59			
ICC(A,1) (95% CI)	0.918 (0.852 to 0.954)	0.898 (0.834 to 0.938)	—
SEM	5.83	6.57	—
MDC_95_	16.15	18.22	—
Quadratic weighted κ (95% CI)	—	—	Sleep: 0.723 (0.575 to 0.828)
Linear weighted κ	—	—	Sleep: 0.553
Exact agreement, %	—	—	Sleep: 49.2

The CFA was estimated using ordinal WLSMV with all 23 physical and psychosocial items entered simultaneously in a correlated two-factor model. Internal consistency coefficients are not applicable to the single-item sleep measure. SEM was calculated as baseline S×√(1−ICC), and MDC_95_ as 1.96×√2×SEM.

CFA, confirmatory factor analysis; CFI, Comparative Fit Index; ICC(A,1), two-way absolute-agreement single-measurement intraclass correlation coefficient; MDC_95_, minimal detectable change at the 95% confidence level; RMSEA, root mean square error of approximation; SEM, SE of measurement; SRMR, standardised root mean square residual; TLI, Tucker-Lewis Index; T-YDQ, Turkish version Young Disability Questionnaire; WLSMV, weighted least squares mean-adjusted and variance-adjusted estimation.

All 23 items loaded significantly on their prespecified factors; standardised loadings ranged from 0.692 to 0.819 for physical and 0.657 to 0.765 for psychosocial (online supplemental material 2, table S1). Separate one-factor sensitivity models are reported in online supplemental material 2, table S5.

### Internal consistency and test–retest reliability

Internal consistency was high for both components across Cronbach’s alpha, ordinal alpha and McDonald’s omega (table 2). Corrected item-total correlations were consistently moderate to strong, and deletion of individual items did not improve Cronbach’s alpha (online supplemental file 2material 2, table S2).

Test–retest reliability was high for both components (ICC(A,1)=0.918 for physical and 0.898 for psychosocial; table 2). The mean difference (T1−T2) was −2.97 points for physical and 0.76 points for psychosocial; the physical score showed a small increase at T2 (p=0.003), whereas psychosocial scores showed no systematic change (p=0.738). Additional agreement analyses are provided in online supplemental material 2, table S4 and online supplemental material 2, figures S1A,B.

For the standalone sleep item, quadratic-weighted kappa was 0.723 (95% CI 0.575 to 0.828); linear-weighted kappa and exact agreement are shown in table 2. No internal-consistency coefficient was calculated.

### Construct validity

Construct validity was supported in the 99 participants who completed both the T-YDQ-spine and SRS-22. Correlations were consistently negative and followed the expected conceptual pattern, with stronger associations between related domains and weaker associations with satisfaction with management (table 3).

**Table 3:** Construct validity of the T-YDQ-spine against SRS-22 domains (n=99)

SRS-22 domain	Physical component, ρ (95% CI)	Psychosocial component, ρ (95% CI)	Sleep item, ρ (95% CI)
Pain	−0.749 (−0.823 to −0.644)	−0.522 (−0.654 to −0.368)	−0.420 (−0.581 to −0.232)
Function/activity	−0.769 (−0.844 to −0.662)	−0.524 (−0.672 to −0.350)	−0.428 (−0.584 to −0.250)
Self-image	−0.379 (−0.534 to −0.202)	−0.634 (−0.743 to −0.493)	−0.413 (−0.559 to −0.246)
Mental health	−0.510 (−0.649 to −0.339)	−0.727 (−0.815 to −0.609)	−0.556 (−0.674 to −0.386)
Satisfaction with management	−0.233 (−0.406 to −0.033)	−0.245 (−0.431 to −0.044)	−0.225 (−0.411 to −0.021)

Values are Spearman’s ρ with 95% CIs. Higher T-YDQ-spine scores indicate greater disability, whereas higher SRS-22 scores indicate better health status; therefore, negative correlations were expected. Seven of eight predefined construct-validity hypotheses were confirmed (87.5%), exceeding the prespecified 75% criterion.

SRS-22, Scoliosis Research Society-22; T-YDQ, Turkish version Young Disability Questionnaire.

Seven of the eight predefined construct-validity hypotheses were confirmed (87.5%), exceeding the prespecified 75% criterion. The only unconfirmed hypothesis involved the sleep item, whose correlation with satisfaction with management was weaker than expected (online supplemental material 2, table S3).

### Floor and ceiling effects

No floor or ceiling effects were observed for the physical or psychosocial components. A floor effect was observed for the sleep item (23.1% at the minimum); the ceiling proportion was 14.6%, below the prespecified threshold (table 4).

**Table 4:** Score distribution, floor and ceiling effects and Section 2 impact items

A T-YDQ-spine score distribution and floor/ceiling effects				
Measure	Median (IQR)	Observed range	Floor, %	Ceiling, %
Physical component (0–100)	36.5 (19.2–50.0)	0–94.2	1.5	0
Psychosocial component (0–100)	42.5 (23.8–56.3)	0–97.5	0.5	0
Sleep item (0–4)	2 (1–3)	0–4	23.1	14.6

B Section 2 impact items			
Section 2 item	Median (IQR)	Observed range	ICC(A,1) (95% CI)
Pain	4 (3–5)	0–10	0.823 (0.718 to 0.891)
Physical activities	4 (3–6)	0–8	0.864 (0.782 to 0.917)
Social relations	4 (3–6)	0–8	0.813 (0.705 to 0.884)
Mood and concentration	4 (3–6)	0–9	0.864 (0.783 to 0.917)

A floor or ceiling effect was defined as ≥15% of participants obtaining the minimum or maximum possible score. Section 2 items are scored individually and are not combined into a summary score.

ICC(A,1), two-way absolute-agreement single-measurement intraclass correlation coefficient; T-YDQ, Turkish version Young Disability Questionnaire.

### Participant prioritisation of impact areas (Section 2)

Median Section 2 scores were 4 for all four impact items, with no missing responses; the observed ranges, IQRs and the high test–retest reliability of individual items (ICC(A,1)=0.813–0.864) are detailed in table 4. Additional measurement-error estimates are reported in online supplemental material 2, table S4. Section 2 items were interpreted individually and not combined into a summary score; their baseline profile is shown descriptively in online supplemental material 2, figure S2.

## Discussion

This study evaluated the cross-cultural adaptation and psychometric properties of the Turkish YDQ-spine in children aged 9–12 years with spinal pain. The original correlated two-factor structure, comprising physical and psychosocial components, showed very good fit; both components demonstrated high internal consistency and test–retest reliability; and seven of eight predefined construct-validity hypotheses against the SRS-22 were confirmed. No relevant floor or ceiling effects were observed for either component, although the standalone sleep item showed a floor effect. Overall, these findings support the structural validity, reliability and construct validity of the T-YDQ-spine in this clinical sample.

The structural findings support the organisation proposed during development of the original instrument. The Danish field-testing study retained 13 physical and 10 psychosocial items, with sleep scored separately and identified reliability, validity, responsiveness and score interpretation as priorities for further research.^8^ In the present study, all 23 component items loaded significantly on their prespecified factors, while the moderate correlation between the latent factors supports related but distinguishable dimensions of spinal pain-related disability. However, this should not be interpreted as structural equivalence between Turkish and Danish children, which would require direct cross-cultural measurement-invariance testing. Internal consistency exceeded 0.90 for both components across all three indices and was comparable to the original development values of 0.90 and 0.84.^8^ At the time of writing, the developer’s register listed published Danish and English versions only, with Hungarian, Turkish and French adaptations under development^29^; therefore, direct comparison with another adapted YDQ-spine version is not yet possible.

Related paediatric measures provide useful context without allowing direct psychometric comparison. The Young Spine Questionnaire, developed for a similar age group, has been adapted into German, Portuguese and Italian^30–33^ with generally satisfactory but variable reproducibility across items and outcomes. The Functional Disability Inventory, a generic paediatric disability measure, has also been used in adolescents with chronic spinal pain,^34 35^ and more recently adapted into Turkish.^36^ These instruments differ from the YDQ-spine in item content, response format and scoring, so their reliability estimates are not directly comparable. Importantly, the YDQ-spine is spinal-specific, age-specific and derived from consequences identified by children themselves,^6^ whereas generic disability measures were not developed around spinal pain and the Young Spine Questionnaire primarily characterises pain rather than its consequences.^30^

Test–retest reliability of both T-YDQ-spine components was high. In addition to ICCs, SEM and MDC_95_ were calculated, with MDC_95_ values of 16.15 points for the physical and 18.22 points for the psychosocial component on 0–100 scales. Reporting measurement error complements relative reliability coefficients because an ICC describes the ability of a measure to distinguish between individuals, whereas SEM and MDC_95_ provide information about the magnitude of score variation attributable to measurement error. For context, the FDI showed internal consistency coefficients of 0.86–0.91 and a 2-week child-report test–retest coefficient of 0.74 in a large paediatric chronic pain sample.^37^ More contemporary paediatric PROM frameworks, including Patient-Reported Outcomes Measurement Information System (PROMIS), increasingly emphasise measurement precision and SE across the score continuum rather than relying solely on conventional reliability coefficients.^38^ Thus, SEM and MDC_95_ provide useful interpretive information for repeated T-YDQ-spine measurements, although clinically important change remains to be established. Despite the high ICC, the physical component showed a small systematic increase at T2 (mean T1−T2 difference, −2.97 points; p=0.003). This modest shift may reflect a retest or familiarity effect, short-term fluctuation in physical symptoms, or, despite the 5-day interval, some influence of repeated administration. Its magnitude was small relative to the MDC95 of 16.15 points; nevertheless, repeated physical scores should be interpreted with awareness of this small systematic retest effect.

Construct validity was supported through predefined hypothesis testing rather than interpretation of correlation magnitudes alone. As expected, the physical component was most strongly associated with SRS-22 function/activity and pain, whereas the psychosocial component showed stronger associations with mental health and self-image; correlations with satisfaction with management were weaker. Seven of eight hypotheses (87.5%) were confirmed, exceeding the 75% threshold considered supportive within Consensus-based Standards for the selection of health Measurement Instruments (COSMIN) methodology.^25^ The single unconfirmed hypothesis involved sleep and satisfaction with management, plausibly because satisfaction with care represents a construct distinct from spinal pain-related health status. The standalone sleep item also requires cautious interpretation: it showed a floor effect, moderate-to-substantial reproducibility by quadratic-weighted kappa,^24^ and lower exact agreement than the multi-item components. This accords with the original field test, in which sleep loaded on both factors and was retained separately.^8^ As a single ordinal item, it should therefore be interpreted as a descriptive indicator rather than a third subscale with equivalent psychometric status.

Section 2 captured the importance children assigned to pain, physical activities, social relations and mood/concentration. All four items showed variability and high individual test–retest reliability, but the study assessed only score distributions and reproducibility. It did not test whether use of these items improves clinical decision-making, facilitates treatment individualisation or leads to better outcomes. They should therefore be considered a potentially useful child-centred adjunct rather than an established intervention-tailoring tool.

The study has several strengths. The Turkish adaptation followed a structured cross-cultural adaptation process, and pretesting was performed in children from the target age group before the final version was used in the main sample. Psychometric evaluation included an ordinal correlated two-factor CFA, multiple indices of internal consistency, absolute-agreement test–retest reliability, measurement-error estimates and hypothesis-based construct-validity testing. The component scores were also recalculated from item-level data and checked against the stored scores before analysis.

### Limitations

This study has several limitations. Participants were recruited from a single tertiary paediatric orthopaedics centre in Istanbul, which may restrict generalisability to community populations and children from other regions of Türkiye. Detailed clinical characteristics, including pain localisation, intensity and duration beyond the minimum 1-week eligibility criterion, were not systematically collected, preventing evaluation of measurement properties across pain phenotypes or severity levels. In addition, the SRS-22 was developed primarily for scoliosis and spinal deformity and is not an age-specific criterion measure for non-specific spinal pain in children. Although several domains were conceptually relevant comparators for the physical and psychosocial components, this constrains interpretation of construct validity.

Test–retest reliability was assessed over a short 5-day interval, and responsiveness to clinical change, longitudinal interpretability and minimal clinically important change were not evaluated. Finally, although the correlated two-factor structure of the Turkish version was supported, cross-cultural measurement invariance between the Turkish and original Danish versions was not tested; direct score equivalence across cultural or language groups therefore cannot be inferred.

## Conclusion

The Turkish version of the YDQ-spine demonstrated evidence of structural validity, high internal consistency and test–retest reliability and supported construct validity in Turkish-speaking children aged 9–12 years with spinal pain. The physical and psychosocial components retained the expected correlated two-factor structure, while the standalone sleep item should be interpreted with greater caution because of its floor effect and single-item format. These findings support the T-YDQ-spine as a promising child-reported measure of spinal pain-related disability in this population. Further multicentre studies are needed to evaluate responsiveness, longitudinal interpretability and cross-cultural measurement invariance.

## Supplementary material

10.1136/bmjopen-2026-120763 online supplemental file 1

10.1136/bmjopen-2026-120763 online supplemental file 2

## Data Availability

Data are available upon reasonable request.
