# Supplementary material for: Cross-cultural adaptation of the Young Disability Questionnaire-Spine into Turkish for children with spinal pain: a methodological validation study
Source: BMJ Open. 2026 Oct 9;16(10):e120763. doi: 10.1136/bmjopen-2026-120763 (PMC13656112; doi:10.1136/bmjopen-2026-120763)
Supplement: Supplementary file 1 [file bmjopen-16-10-s001.pdf]

Genç Engellilik Anketi

Sürüm: 9 – 12 yaş

Türkçe

Boyun, sırt veya belindeki ağrı seni nasıl etkiliyor?

Aşağıdaki soruları yanıtlarken, ağrının seni nasıl etkilediğini düşünmen önemlidir. Sorularda yazan şeyi hiç hissetmediysen, “Hiç” kutusuna bir (X) işareti koy. Her soruya yalnızca bir yanıt vermelisin.

		Hiç	Bir iki kez	Bazen	Sıklıkla	Çoğu zaman
1.	Ağrım olduğunda kendi bakımımı yapmakta zorlanırım (örneğin giyinmek, duş almak veya banyo yapmak gibi)	☐	☐	☐	☐	☐
2.	Ağrım olduğunda başım öne eğik oturmakta zorlanırım (telefonuma bakmak veya ödevimi yapmak)	☐	☐	☐	☐	☐
3.	Ağrım olduğunda ağır eşyaları kaldırmakta zorlanırım	☐	☐	☐	☐	☐
4.	Ağrım olduğunda eğilmekte zorlanırım	☐	☐	☐	☐	☐
5.	Ağrım olduğunda, normalde yapabildiğim süre kadar ayakta durmakta zorlanırım	☐	☐	☐	☐	☐
6.	Ağrım olduğunda, normalde yapabildiğim süre kadar oturmakta zorlanırım	☐	☐	☐	☐	☐
7.	Ağrım olduğunda, normalde yürüyebildiğim kadar yürümekte zorlanırım	☐	☐	☐	☐	☐
8.	Ağrım olduğunda, normalde koşabildiğim kadar iyi koşamam	☐	☐	☐	☐	☐
9.	Ağrım olduğunda, normalde yaptığım gibi spor yapamam	☐	☐	☐	☐	☐
10.	Ağrım olduğunda, normalde yapabildiğim kadar oyun oynamakta zorlanırım	☐	☐	☐	☐	☐
11.	Ağrım olduğunda bisiklet sürmekte zorlanırım	☐	☐	☐	☐	☐

		Hiç	Bir iki kez	Bazen	Sıklıkla	Çoğu zaman
12.	Ağrım olduğunda, normalde olduğu kadar eğlenemiyorum	☐	☐	☐	☐	☐
13.	Ağrım olduğunda, normalden daha yalnız hissederim	☐	☐	☐	☐	☐
14.	Ağrım olduğunda endişeleniyorum	☐	☐	☐	☐	☐
15.	Ağrım olduğunda korkarım	☐	☐	☐	☐	☐
16.	Ağrım olduğunda, daha kolay kızıyorum veya sinirleniyorum	☐	☐	☐	☐	☐
17.	Ağrım olduğunda, daha kolay üzülürüm veya hüzünlenirim	☐	☐	☐	☐	☐
18.	Ağrım olduğunda konsantre olmakta zorlanıyorum	☐	☐	☐	☐	☐
19.	Ağrım olduğunda okula gitmem, evde kalırım	☐	☐	☐	☐	☐
20.	Ağrım olduğunda iyi uyuyamıyorum	☐	☐	☐	☐	☐
21.	Ağrım olduğunda enerjim azalır	☐	☐	☐	☐	☐
22.	Ağrım olduğunda, annem ve babamla bunun hakkında konuşmakta zorlanırım	☐	☐	☐	☐	☐
23.	Ağrım olduğunda, arkadaşlarımla bunun hakkında konuşmakta zorlanırım	☐	☐	☐	☐	☐
24.	Ağrım olduğunda tedavi alırım (örneğin, doktor veya fizyoterapistten)	☐	☐	☐	☐	☐

Sonraki 4 soru için, aşağıdaki örnekte olduğu gibi bir kutucuğu işaretleyiniz:

Hiç önemli değil — Çok önemli

0	1	2	3	4	5 (X)	6	7	8	9	10

Boyun, sırt veya belimdeki ağrının en kötü yanı...

a. Çok fazla acı veriyor olması

Hiç önemli değil — Çok önemli

0	1	2	3	4	5	6	7	8	9	10

b. Dışarıda istediğim kadar rahat hareket edemiyor olmak

Hiç önemli değil — Çok önemli

0	1	2	3	4	5	6	7	8	9	10

c. Arkadaşlarımla fazla vakit geçiremiyor olmak

Hiç önemli değil — Çok önemli

0	1	2	3	4	5	6	7	8	9	10

d. Üzgün veya sinirli olmak ve konsantre olamamak

Hiç önemli değil — Çok önemli

0	1	2	3	4	5	6	7	8	9	10
