# Supplementary material for: Cross-cultural adaptation of the Young Disability Questionnaire-Spine into Turkish for children with spinal pain: a methodological validation study
Source: BMJ Open. 2026 Oct 9;16(10):e120763. doi: 10.1136/bmjopen-2026-120763 (PMC13656112; doi:10.1136/bmjopen-2026-120763)
Supplement: Supplementary file 2 [file bmjopen-2026-120763.reviewer_comments.pdf]

PEER REVIEW HISTORY

BMJ Open publishes all reviews undertaken for accepted manuscripts. Reviewers are asked to complete a checklist review form and are provided with free text boxes to elaborate on their assessment. These free text comments are reproduced below.

ARTICLE DETAILS

Title (Provisional)

Cross-cultural adaptation of the Young Disability Questionnaire–Spine into Turkish for children with spinal pain: a methodological validation study

Authors

iyigün, abdullah; günerbüyük, caner; demir, pervin; polat, ömer; güner, kürşat; aksekili, mehmet atıf

---

VERSION 1 - REVIEW

**Reviewer**	**1**
**Name**	**Kuru Çolak , Tuğba**
**Affiliation**	**Marmara University**
**Date**	**13-May-2026**
**COI**	**None**

Thank you for your kind invitation to review this manuscript.

The manuscript aims to examine the cross-cultural adaptation, validity, and reliability of the Turkish version of the Young Disability Questionnaire–Spine in children aged 9–12 years with spinal pain. I think that the topic is important, because spinal pain in children may be more common than generally assumed and may negatively affect daily living activities, school participation, and social life. Therefore, introducing valid and reliable age-specific assessment tools into Turkish is valuable for both clinical practice and research. The sample size of the study generally appears to be adequate, and the fact that the translation and cross-cultural adaptation process was conducted according to the recommendations of Beaton et al. is one of the strengths of the study. In addition, the assessment of internal consistency, test-retest reliability, CFA, convergent validity, and floor/ceiling effects indicates that the study provides a comprehensive psychometric evaluation.

My suggestions regarding the manuscript are presented below.

Title

The title is generally appropriate and reflects the aim of the study.

Abstract

The abstract generally reflects the content of the manuscript. However, the statement “Strong negative correlations were observed between T-YDQ-spine and SRS-22 scores (r = –0.90 to –0.99)” is noteworthy. Such high correlation values may raise questions for readers regarding the analysis, scoring direction, or data entry. Therefore, I recommend that the authors explain this finding in more detail in the main text and, if necessary, state that the analyses were rechecked.

Keywords

I recommend that the keywords be selected in accordance with MeSH terms.

Introduction

The Introduction section is generally understandable and presents the rationale of the study. Mentioning the effects of spinal pain in children on daily life, school absenteeism, and social participation supports the importance of the study. However, in the Introduction, it could be emphasized more clearly why the YDQ-spine is more appropriate for this age group compared with other scales. In addition, it is stated that this study is the second cross-cultural adaptation study of the YDQ-spine; since this is important information, it should be more strongly linked to the gap in the literature.

In the first paragraph, the frequency of pain is emphasized; however, I recommend that the possible causes of spinal pain also be briefly mentioned.

Methods

The inclusion of 199 children aged 9–12 years with spinal pain appears to be appropriate in terms of sample size. However, the clinical characteristics of the participants, such as the type of spinal pain complaints, pain localization, pain duration, and pain intensity, should be reported in more detail. This information would contribute to a better understanding of the sample and to the evaluation of the generalizability of the findings.

Please clarify for readers which scale includes 13 items and which scale includes 10 items.

The structure of the YDQ-spine is presented somewhat inconsistently in the text. It should be stated more clearly how many items Section 1 includes in total, how many items are included in the Physical component, how many items are included in the Psychosocial component, and how the Sleep item is evaluated. In addition, the role of Section 2 in the psychometric analysis and its purpose in clinical use should be explained more clearly. This section is important for readers to understand the scoring and interpretation structure of the scale.

It is not mentioned whether permission was obtained from the developers of the questionnaire during the translation and cross-cultural adaptation process. Forward translation, synthesis, back-translation, expert committee review, and pretesting with 15 children are positive aspects. However, the feedback received from the children during pretesting and the minor linguistic modifications that were made could be presented in more detail. This information would increase readers’ confidence in the cultural appropriateness of the Turkish form.

Were there any words or sentences that needed to be changed?

A 5-day interval was used for test-retest reliability. This interval may be appropriate; however, the reason for choosing 5 days should be justified with a short sentence. For example, it may be stated that this interval was chosen to reduce recall bias while limiting the possibility of clinical change. In addition, it should be explained more clearly how the 59 participants selected for the test-retest assessment were randomly chosen.

Furthermore, selecting 59 participants appears to be inconsistent with the aim of including 10 participants per item.

It is stated that a polychoric correlation matrix was used in the CFA analysis. This is an appropriate approach for ordinal data. However, the estimator used in the CFA is not clearly specified. If WLSMV, DWLS, or another estimator was used, this should be clearly stated in the Methods section. This is important for the reproducibility of the results.

The SRS-22 was used for convergent validity. This scale is mainly used in the field of scoliosis and spinal deformity. The present study, however, includes children aged 9–12 years with spinal pain. Therefore, the authors should provide a stronger justification for why the SRS-22 was selected as an appropriate comparator for convergent validity in this age group and clinical context.

I also recommend that the SRS-22 be briefly explained in this section and that a reference be added.

Results

The CFA results appear to be very good. However, the fact that some values are very high, particularly the reporting of CFI as 1.000 and TLI as 1.001, makes it necessary to state the estimator and modelling approach more clearly. These results may be correct; however, more detailed information should be provided in the Methods section so that readers can clearly understand the analysis process.

The internal consistency and test-retest results are strong. Cronbach's alpha was reported as 0.912 for the Physical component and 0.887 for the Psychosocial component, and the ICC values were also quite high. These findings support the reliability of the scale. However, the SEM formula in the table note appears to be incorrect or incomplete. The standard SEM formula is generally given as SD × √(1 – ICC). I recommend that the table note be checked in this regard.

The convergent validity findings are noteworthy. Very high negative correlations ranging from r = –0.90 to –0.99 were reported between the YDQ-spine and the SRS-22. Although these values are in the expected direction, they are quite high for psychometric studies. I recommend that the authors recheck the scoring direction, data entry, and analyses, and discuss the possible reasons for these high correlations. It would also be useful to explain whether such high correlations indicate that the two scales measure very similar constructs or whether they may be related to a methodological issue.

It is positive that floor and ceiling effects were analyzed. The presence of a floor effect for the Sleep item may be a reasonable finding; however, it should be emphasized more clearly in the Discussion that the single-item structure may limit variability and that the Sleep item should therefore be interpreted with caution.

I recommend that the data presented in the tables not be repeated in the text in the same way.

Discussion

The Discussion section generally summarizes the findings. However, in some parts, the results are interpreted somewhat strongly. For example, expressions such as “excellent validity” and “structural equivalence” should be used more cautiously. This study supports the structural validity of the Turkish version; however, since cross-cultural measurement invariance was not tested, the term “structural validity” would be more appropriate than “structural equivalence.”

Since there are no other cross-cultural adaptation studies on the YDQ-spine, it is understandable that the authors refer to studies on the Young Spine Questionnaire. However, it should be stated more clearly that these two scales are not the same measurement tool, and the comparisons should be made more cautiously. In particular, expressions such as “our results exceeded…” may imply direct superiority; therefore, a more neutral wording should be preferred.

The limitation related to the use of the SRS-22 for convergent validity could also be added to the limitations section. In addition, the fact that the sample was recruited only from a tertiary center in Istanbul may limit the generalizability of the findings to the general child population in Türkiye.

The Turkish form presented as supplementary material is generally understandable. However, some expressions could be reconsidered in terms of child-friendly language. For example, the expression “Ağrım olduğunda okula gitmem evde kalırım” could be written more clearly as “Ağrım olduğunda okula gitmem, evde kalırım” or “Ağrım olduğunda okula gitmek yerine evde kalırım.” In addition, the word “konsantre olamamak” appears to be divided in the visual presentation in the final section; therefore, the form should be checked carefully in terms of formatting in the final version.

Although the general structure of the manuscript is understandable, it requires comprehensive revision in terms of English grammar and expression. For example, expressions such as “patients who applied to the at a tertiary pediatric orthopedics center,” “Internal consistency is evaluate,” “cross cultural adaptation studies … is already under development,” and “5 days interval” should be corrected. I recommend that the manuscript undergo professional English language editing before publication.

Reviewer **2**

Name **Otani, Koji**

Affiliation **Fukushima Medical Univ., School of Medicine, Dept. of Orthopaedic Surgery**

Date **11-Aug-2026**

COI **None**

The manuscript titled "Cross-cultural adaptation, validity, and reliability of the Turkish version of the Young Disability Questionnaire-Spine for children with spinal pain" is considered suitable for

publication. The content demonstrates that the necessary procedures for translating a questionnaire developed in a foreign language into the target language were appropriately followed. However, revisions are required due to several unresolved issues regarding the analysis methods.

1. The authors should clarify both the source version and the authorization process for the Turkish adaptation of the YDQ-spine. Please specify the version/date of the English questionnaire that was used for translation. In addition, since the official YDQ-spine website states that translation or modification of the instrument requires permission from the developers, the authors should report whether permission for the Turkish translation was obtained and whether the final Turkish version—including its wording, instructions, response options, and layout—was reviewed and approved by the original developers. This is particularly relevant because the wording and anchors of the final four "what matters most" items appear to correspond to an earlier version of the YDQ-spine rather than the currently available English version. Clarification of these points is important for documenting the fidelity and reproducibility of the cross-cultural adaptation process.

2. The structural validity analysis requires clarification. Since the original YDQ-spine established a two-factor structure consisting of 13 Physical and 10 Psychosocial items, CFA is an appropriate approach for the Turkish adaptation. However, the manuscript reports separate fit indices for the Physical and Psychosocial components, suggesting that two separate one-factor CFAs may have been conducted. If so, these analyses assess the unidimensionality of the two subscales separately but do not, by themselves, confirm the original two-factor structure. The authors should therefore fit all 23 items simultaneously in a correlated two-factor CFA model. If such a model has already been fitted, the overall model-fit indices and the latent correlation between the Physical and Psychosocial factors should be reported. In addition, the estimator used for the ordinal items (e.g., WLSMV or DWLS), the method used to obtain the polychoric correlations, the handling of missing data, and the item-level standardized factor loadings should also be reported.

3. Page 6, Line 41-43; "Responses spanned the full scale for all questions, with minimum scores of 0.0 and maximum scores of 10.0, indicating that the clinician or researcher can identify which impact areas are most relevant for each child and tailor intervention strategies accordingly."

The statement that the observed 0–10 score range enables clinicians to identify the most relevant impact areas and tailor interventions seems to go beyond the data presented. The score distribution demonstrates variability, but clinical utility for prioritizing treatment was not evaluated. Please consider moderating this interpretation or framing it as a potential clinical application rather than a study finding.

4. Please clarify the ICC methodology used for the four Section 2 impact questions (YDQ_A–D). The manuscript reports excellent test–retest reliability based on ICCs, but does not specify the ICC model, whether absolute agreement or consistency was assessed, or whether single- or average-measure ICCs were calculated. Please also indicate whether the same two-way mixed, absolute-agreement model described for Section 1 was used for these items.

VERSION 1 - AUTHOR RESPONSE

Reviewer 1

We thank Reviewer 1 for the detailed and constructive comments.

Abstract

Comment: The very high negative correlations between T-YDQ-spine and SRS-22 should be rechecked and explained.

Response: We agree that the originally reported correlations were unexpectedly high and warranted careful verification. During data verification, data-entry errors were identified in the previously entered dataset. The item-level data were therefore re-entered from the original source records, and all psychometric analyses were rerun from scratch using the re-entered dataset. The revised construct-validity coefficients were substantially lower and showed a differentiated, conceptually plausible pattern across SRS-22 domains. To strengthen the construct-validity assessment, we also replaced interpretation based solely on correlation magnitude with a predefined hypothesis-testing framework. Seven of eight hypotheses (87.5%) were confirmed.

Change in manuscript: The Abstract, Statistical Analysis, Results, Table 3, Discussion and Online Supplemental Table S3 were revised to reflect the corrected construct-validity analysis.

Keywords

Comment: Keywords should be selected according to MeSH terms.

Response: We agree. The keywords were revised using MeSH-compatible terminology.

Change in manuscript: The revised keywords are: “Back Pain; Child; Surveys and Questionnaires; Reproducibility of Results; Cross-Cultural Comparison.”

Introduction

Comment: Please explain more clearly why the YDQ-spine is particularly appropriate for this age group and strengthen the literature-gap rationale.

Response: We agree. The Introduction now emphasises that the YDQ-spine was specifically developed for children aged 9–12 years and was based on consequences of spinal pain identified as important by children themselves. We also clarified the absence of a validated Turkish instrument specifically assessing spinal pain-related disability in this age group. During revision, we removed the previous statement describing the present study as the “second cross-cultural adaptation,” because the available information did not support retaining a precise numerical claim.

Change in manuscript: The Introduction now describes the age-specific and child-centred development of the instrument and focuses on the clinically relevant gap: no validated Turkish YDQ-spine is currently available for children aged 9–12 years with spinal pain.

Comment: Possible causes of spinal pain should also be briefly mentioned.

Response: We agree. A brief statement describing the multifactorial nature of spinal pain in childhood has been added.

Change in manuscript: The Introduction now states that childhood spinal pain may reflect biological, behavioural, psychosocial and environmental influences.

Methods — participant characteristics

Comment: More detailed clinical characteristics such as pain localisation, duration and intensity should be reported.

Response: We agree that these data would improve clinical characterisation of the cohort. However, pain localisation, pain intensity and detailed duration beyond the minimum 1-week eligibility criterion were not systematically collected and therefore could not be reported retrospectively. We have stated this explicitly and have also acknowledged the resulting limitation regarding subgroup

analyses and generalisability.

Change in manuscript: This information has been added to both Study Design and Participants and Limitations.

Structure of the YDQ-spine

Comment: Please clarify which component contains 13 items and which contains 10 items, how many items are included in Section 1, how Sleep is evaluated, and the role of Section 2.

Response: We agree and have substantially clarified the instrument structure. Section 1 contains 24 items: 13 Physical items, 10 Psychosocial items and one independently scored Sleep item. Physical and Psychosocial components are transformed separately to 0–100 scores and no combined total score is calculated. Section 2 comprises four optional 0–10 impact items that are scored individually and are not incorporated into Section 1 scores.

Change in manuscript: The Young Disability Questionnaire–Spine subsection now provides the complete structure, scoring and interpretation of Sections 1 and 2.

Permission and pretesting

Comment: Please clarify whether developer permission was obtained and provide more detail on feedback from the 15 children and linguistic modifications.

Response: Permission was obtained from the developer before translation began. We have also added the specific wording changes made after pretesting. In item 17, “get upset” was initially translated as “kederlenirim” and subsequently changed to “hüzünlenirim” because the latter wording was more readily understood by the children. In item 24, “chiropractor” was initially translated as “kayropraktör” and was replaced with “masör” because several children were unfamiliar with the former term. Following completion of the adaptation process, the final Turkish version and translation documentation were submitted to the developer and received formal approval.

Change in manuscript: These details have been added to the Translation and Cross-Cultural Adaptation section.

Test–retest interval and participant selection

Comment: Please justify the 5-day interval and explain how the 59 participants were randomly selected.

Response: The 5-day interval was chosen to reduce recall of previous responses while limiting the likelihood of substantial clinical change between assessments. We would also like to clarify that the test–retest participants were not randomly selected. The first 59 participants enrolled in the study constituted the planned test–retest subsample. We have revised the manuscript to state this explicitly.

Change in manuscript: The Study Design and Participants and Statistical Analysis sections now state that the first 59 participants completed the questionnaire again after 5 days and provide the rationale for this interval.

Sample size

Comment: Selecting 59 participants appears inconsistent with the aim of including 10 participants per item.

Response: We appreciate this observation. The original wording did not distinguish sufficiently between sample-size requirements for different psychometric analyses. Sample sizes for structural validity, test–retest reliability and construct validity were planned separately before recruitment. The full sample of 199 participants was used for CFA, 59 participants for test–retest reliability and 99 for construct validity. For CFA, we no longer rely on a fixed participant-per-item rule; instead, sample adequacy is justified using simulation evidence indicating that requirements depend on model complexity, number of indicators and magnitude of factor loadings.

Change in manuscript: A dedicated sample-size paragraph has been added to Study Design and Participants.

CFA estimator

Comment: The estimator used for CFA should be clearly specified.

Response: We agree. All 23 Physical and Psychosocial items were entered simultaneously into a correlated two-factor CFA and treated as ordered categorical indicators. Weighted least squares mean- and variance-adjusted estimation (WLSMV) based on polychoric correlations was used.

Change in manuscript: The CFA model, estimator, handling of ordinal data, fit indices and missing-data approach are now specified in detail in Statistical Analysis.

SRS-22 as comparator

Comment: Please provide a stronger justification for using the SRS-22 and briefly explain the instrument.

Response: We agree. The SRS-22 was selected because several of its domains—particularly Pain, Function/Activity, Mental Health and Self-image—have conceptual overlap with the Physical and Psychosocial consequences assessed by the YDQ-spine. However, we acknowledge that the SRS-22 was developed primarily for scoliosis and spinal deformity and is not an age-specific criterion instrument for nonspecific spinal pain. Accordingly, we now describe it as an external comparator for hypothesis-based construct validity rather than as a criterion standard.

Change in manuscript: The rationale and limitations of the SRS-22 are now stated in the Measurement Tools section and discussed explicitly under Limitations.

CFA results

Comment: Very high CFI and TLI values require clearer description of the estimator and modelling approach.

Response: We agree. The structural model was rerun as a simultaneous correlated two-factor ordinal CFA using WLSMV. The revised model yielded CFI=0.997, TLI=0.996, RMSEA=0.018 and SRMR=0.045. The latent correlation between Physical and Psychosocial factors was 0.586.

Change in manuscript: The revised modelling approach is described in Statistical Analysis, and the overall fit indices are reported in Results and Table 2. Item-level loadings are provided in Online Supplemental Table S1.

SEM formula and reliability statistics

Comment: The SEM formula should be checked.

Response: We agree. SEM was recalculated and is now explicitly defined as baseline SD × $\sqrt{(1-ICC)}$. We additionally report MDC_{95}, calculated as 1.96 × $\sqrt{2}$ × SEM. We also examined systematic change between administrations. A small but statistically significant increase was observed in the Physical component at retest (T1−T2 mean difference −2.97, Wilcoxon $p=0.003$), whereas no systematic change was observed for the Psychosocial component.

Change in manuscript: The formulas are specified in the Statistical Analysis section and in the footnote to Table 2. SEM and MDC_{95} are reported for both Physical and Psychosocial components.

Construct validity correlations

Comment: The very high correlations should be rechecked and their implications discussed.

Response: As noted above, the data, scoring and analyses were rechecked and the construct-validity analysis was repeated using the corrected dataset. The revised correlations are substantially lower than those originally reported and display the expected conceptual pattern. Rather than treating individual correlation magnitude as evidence of validity, we now use eight predefined hypotheses addressing expected direction, magnitude and relative strength of associations. Seven of eight hypotheses were confirmed.

Change in manuscript: The revised analysis is reported in Statistical Analysis, Construct Validity, Table 3 and Online Supplemental Table S3, and its interpretation has been revised in the Discussion.

Sleep floor effect

Comment: The single-item structure of Sleep may limit variability and should be interpreted cautiously.

Response: We agree. The Sleep item showed a floor effect, with 23.1% of participants selecting the minimum response category. We now explicitly emphasise that, because Sleep is represented by a single ordinal item, its ability to capture variability is more limited than that of the multi-item components and it should not be interpreted as a psychometrically equivalent third subscale.

Change in manuscript: This interpretation has been added to the Discussion and reflected in the Conclusion.

Repetition of tables in text

Comment: Data presented in tables should not be repeated in the text.

Response: We agree. The Results section has been substantially shortened. Numerical detail is now primarily retained in Tables 1–4, while the text focuses on the principal findings and interpretation.

Change in manuscript: The Internal Consistency, Construct Validity, Floor and Ceiling Effects, and Section 2 Results paragraphs were condensed and now direct the reader to the relevant tables and online supplemental material.

Discussion — strength of interpretation

Comment: Terms such as "excellent validity" and "structural equivalence" should be used more cautiously.

Response: We agree. We have removed overstrong terminology. The revised manuscript refers to evidence supporting "structural validity" and explicitly states that similarity of factor structure should not be interpreted as cross-cultural structural equivalence because measurement invariance between the Turkish and Danish versions was not evaluated.

Change in manuscript: The Abstract, Discussion, Limitations and Conclusion were revised accordingly.

Comparison with the Young Spine Questionnaire

Comment: YSQ is a different instrument and comparisons should be more cautious; superiority should not be implied.

Response: We agree. The YSQ literature is now used only to provide contextual information from paediatric spinal PROM research. We explicitly state that the YSQ and YDQ-spine differ in construct, content, response format and scoring, and that their psychometric coefficients should not be directly compared.

Change in manuscript: The relevant Discussion paragraph has been rewritten using neutral contextual wording without claims of superiority.

Limitations

Comment: The limitations of using the SRS-22 and recruiting from a single tertiary centre should be stated.

Response: We agree. Both limitations are now explicitly acknowledged.

Change in manuscript: The Limitations section now discusses the restricted generalisability of the single-centre sample and the limitations of using the SRS-22 outside its primary scoliosis/spinal deformity context.

Turkish supplementary questionnaire

Comment: Please revise the Turkish wording/formatting, including punctuation in the school item and the split word "konsantre olamamak."

Response: We agree. The Turkish questionnaire was reviewed for wording and layout, and the noted punctuation and formatting issues were corrected. The final version was subsequently reviewed and formally approved by the developer.

Change in manuscript: The final T-YDQ-spine is provided as Online Supplemental Material 1, and formal developer approval is documented in the Translation and Cross-Cultural Adaptation section.

English language

Comment: Comprehensive English editing is recommended.

Response: The manuscript has undergone comprehensive language and style revision. Grammar, terminology, abbreviation use and sentence structure were reviewed throughout, and the manuscript was also substantially shortened to reduce redundancy.

Change in manuscript: The manuscript has been revised throughout.

Reviewer 2

We thank Reviewer 2 for the focused methodological comments, which prompted important clarifications and additional analyses.

Comment 1 — Source version and authorization

Comment: Please clarify the source version/date of the English YDQ-spine, permission to translate it, and whether the final Turkish wording, instructions, response options and layout were reviewed and approved by the developers. The Section 2 wording appears to correspond to an earlier English version.

Response: We agree that this requires explicit documentation. Permission to translate and culturally adapt the YDQ-spine was obtained from Henrik Hein Lauridsen on 18 December 2023, and the English version available on that date was used as the source instrument. The developer subsequently revised the English YDQ-spine on 23 February 2024. This change affected only the question stem and response anchors of the four optional Section 2 impact items. The 24-item Section 1 structure, item content and scoring were unchanged. Because the Turkish adaptation had already begun using the December 2023 version, the study retained that source version. Following completion of the adaptation process, the final Turkish questionnaire—including wording, instructions, response options and layout—together with the translation documentation was submitted to the developer and received formal approval.

Change in manuscript: A detailed description has been added to Translation and Cross-Cultural Adaptation, and the final questionnaire is supplied as Online Supplemental Material 1.

Comment 2 — Structural validity

Comment: All 23 Physical and Psychosocial items should be fitted simultaneously in a correlated two-factor CFA. Overall model fit, latent factor correlation, estimator, polychoric correlations, handling of missing data and item-level standardised loadings should be reported.

Response: We agree. The structural validity analysis was rerun using all 23 items simultaneously in the prespecified correlated two-factor model comprising 13 Physical and 10 Psychosocial items. The ordinal items were estimated using WLSMV with polychoric correlations. Pairwise present observations were specified for missing item-level data. The correlated two-factor model showed very good fit: robust $\chi^2(229)=243.37$, $p=0.245$; CFI=0.997; TLI=0.996; RMSEA=0.018 (90% CI 0.000–0.035); and SRMR=0.045. The latent Physical–Psychosocial correlation was 0.586. All 23 items loaded significantly on their prespecified factors. The previously performed separate one-factor models are now retained only as sensitivity analyses.

Change in manuscript: The revised CFA methodology is described in Statistical Analysis; overall results are reported in Structural Validity and Table 2; item-level loadings are provided in Online Supplemental Table S1; and the separate one-factor sensitivity models are presented in Online

Supplemental Table S5.

Comment 3 — Interpretation of Section 2

Comment: The statement that the Section 2 score range allows clinicians to identify relevant areas and tailor interventions goes beyond the data.

Response: We agree. The previous interpretation overstated the clinical implications of the Section 2 findings. The study assessed only score distributions and reproducibility and did not evaluate whether Section 2 improves treatment prioritisation, clinical decision-making or outcomes.

Change in manuscript: The Results now describe Section 2 items only as individual descriptive impact items, and the Discussion explicitly states that they should be viewed as a potentially useful child-centred adjunct rather than an established intervention-tailoring tool.

Comment 4 — ICC methodology for Section 2

Comment: Please specify the ICC model, agreement type and measurement unit for the four Section 2 impact items and clarify whether the same model used for Section 1 was applied.

Response: We agree. The same ICC model was applied to the Physical and Psychosocial component scores and to each of the four Section 2 impact items: a two-way, absolute-agreement, single-measurement ICC, reported as ICC(A,1) in the McGraw and Wong nomenclature and equivalent to ICC(2,1) in the Shrout and Fleiss classification.

Change in manuscript: The ICC methodology is now specified in detail in Statistical Analysis, and Section 2 ICC estimates with 95% CIs are reported in Table 4.

We thank the Editor and both Reviewers for the care taken with this manuscript. Their comments prompted a thorough re-examination of our data, which led us to identify and correct data-entry errors, and their methodological guidance has substantively improved the analysis and the caution with which we interpret it. We are submitting a clean copy of the revised manuscript, a version with all changes marked, Online Supplemental Material 1 (the final Turkish questionnaire) and Online Supplemental Material 2 (supplemental tables and figures). We hope the revised manuscript is now suitable for publication in BMJ Open and would be glad to address any further points.

VERSION 2 - REVIEW

**Reviewer**	**1**
**Name**	**Kuru Çolak , Tuğba**
**Affiliation**	**Marmara University**
**Date**	**17-Sep-2026**
**COI**	

I would like to thank the authors for their extensive and thoughtful revision.

Authors have addressed the reviewers' main methodological concerns, including clarification of the source version and authorization process, re-analysis of structural and construct validity, more cautious interpretation of Section 2, and clearer reporting of reliability and measurement-error analyses.

I think the revised manuscript is substantially improved and is suitable for publication after minor revision.

I have minor comments:

- Although test–retest reliability was high, the Physical component showed a small but statistically significant systematic difference between T1 and T2 (p=0.003). This result is appropriately reported, but a brief discussion of its possible implications, such as a retest or recall effect, would strengthen the interpretation.

- The replacement of "chiropractor" with "masör" should be reconsidered. These terms do not represent equivalent healthcare professions or levels of professional training. A chiropractor is a practitioner with formal university-based professional education in chiropractic, whereas "masör" refers to a different occupation focused on massage practice. This replacement may therefore alter the intended meaning of the item rather than constitute a cultural adaptation. I recommend retaining "kayropraktör," potentially with a brief child-friendly explanatory phrase in parentheses.

VERSION 2 - AUTHOR RESPONSE

We sincerely thank the reviewer for the careful evaluation of our revised manuscript and for the constructive minor comments. We have addressed both points and revised the manuscript and the Turkish questionnaire accordingly. Our point-by-point responses are provided below.

Comment 1

Although test–retest reliability was high, the Physical component showed a small but statistically significant systematic difference between T1 and T2 (p=0.003). This result is appropriately reported, but a brief discussion of its possible implications, such as a retest or recall effect, would strengthen the interpretation.

Response

Thank you for this helpful comment. We agree that the small systematic difference observed for the Physical component warrants further interpretation despite the high test–retest ICC. We have therefore expanded the Discussion to acknowledge that the modest increase in Physical scores at T2 may reflect a retest/familiarity effect, residual recall over the 5-day interval, or natural short-term fluctuation in physical symptoms. We also note that the magnitude of the mean difference (2.97 points on the 0–100 scale) was small relative to the MDC95 (16.15 points), and therefore should not be interpreted as evidence of clinically meaningful change. We have added this clarification to the Discussion.

Text added to the Discussion

"Despite the high ICC, the Physical component showed a small systematic increase at T2 (mean T1−T2 difference, −2.97 points; p=0.003). This modest shift may reflect a retest or familiarity effect, short-term fluctuation in physical symptoms, or, despite the 5-day interval, some influence of repeated administration. Its magnitude was small relative to the MDC95 of 16.15 points; nevertheless, repeated Physical scores should be interpreted with awareness of this small systematic retest effect."

Comment 2

The replacement of "chiropractor" with "masör" should be reconsidered. These terms do not represent equivalent healthcare professions or levels of professional training. A chiropractor is a practitioner with formal university-based professional education in chiropractic, whereas "masör" refers to a different occupation focused on massage practice. This replacement may therefore alter the intended meaning of the

item rather than constitute a cultural adaptation. I recommend retaining "kayropraktör," potentially with a brief child-friendly explanatory phrase in parentheses.

Response

Thank you for this important observation. We agree that "masör" is not professionally or semantically equivalent to "chiropractor," and we have reconsidered the wording of Item 24 accordingly.

However, we were reluctant to reintroduce "kayropraktör" into the Turkish version. During pretesting in children from the target age group, several participants were unfamiliar with this term, which was the reason it was replaced before administration of the questionnaire in the main study. Adding an explanatory phrase would introduce additional wording that was not cognitively pretested and would also increase the linguistic complexity of an item designed for children aged 9–12 years.

Importantly, the construct assessed by Item 24 is whether the child receives treatment when experiencing spinal pain, rather than recognition of any specific healthcare profession. The examples of "doctor" and "physiotherapist" are familiar to Turkish children and adequately illustrate the intended concept of receiving treatment. We therefore considered that the most appropriate solution was to remove "masör" without reintroducing "kayropraktör." The final wording is now: "Ağrım olduğunda tedavi alırım (örneğin, doktor veya fizyoterapistten)."

For transparency, we have clarified in the manuscript that the version administered to the 199 participants contained "masör," as determined after the original pretesting process. The present change was made after data collection in response to the reviewer's valid concern regarding semantic equivalence and affects only one illustrative example; the item stem, response options, scoring and factor assignment are unchanged.

Revised wording of Item 24 in T-YDQ

"Ağrım olduğunda tedavi alırım (örneğin, doktor veya fizyoterapistten)."

Clarification added to the Translation and Cross-Cultural Adaptation section

"In item 24, 'chiropractor', initially translated as 'kayropraktör', was replaced with 'masör' because several children were unfamiliar with the former term. This was the wording administered to the main study sample. During peer review, the use of 'masör' was reconsidered because it is not professionally or semantically equivalent to 'chiropractor'. Rather than reintroducing 'kayropraktör', which had not been understood by several children during pretesting, 'masör' was removed from the illustrative examples in the final publication version, leaving 'doctor' and 'physiotherapist'. The item stem, response options and scoring were unchanged."

We thank the reviewer again for these comments, which helped us improve the clarity and methodological transparency of the manuscript.
