# Supplementary material for: Cross-cultural adaptation of the Young Disability Questionnaire-Spine into Turkish for children with spinal pain: a methodological validation study
Source: BMJ Open. 2026 Oct 9;16(10):e120763. doi: 10.1136/bmjopen-2026-120763 (PMC13656112; doi:10.1136/bmjopen-2026-120763)
Supplement: Supplementary file 3 [file bmjopen-16-10-s002.docx]

**ONLINE SUPPLEMENTAL MATERIAL 2**

**Cross-cultural adaptation of the Young Disability Questionnaire–Spine into Turkish for children with spinal pain: a methodological validation study**

**Contents**

Supplementary Table S1. Standardized factor loadings from the correlated two-factor CFA

Supplementary Table S2. Item-level internal consistency statistics

Supplementary Table S3. Prespecified construct-validity hypotheses and results

Supplementary Table S4. Additional test–retest agreement and measurement-error analyses

Supplementary Table S5. Exploratory sex measurement invariance and sensitivity CFA analyses

Supplementary Figure S1A. Bland–Altman plot for the Physical component

Supplementary Figure S1B. Bland–Altman plot for the Psychosocial component

Supplementary Figure S2. Section 2 impact-item radar plot

# Supplementary Table S1. Standardized factor loadings from the correlated two-factor CFA

| **Factor** | **Item** | **Item label** | **Standardized loading** | **95% CI** |
| --- | --- | --- | --- | --- |
| Physical | Q1 | Take care of myself | 0.757 | 0.687–0.826 |
| Physical | Q2 | Head bend forward | 0.753 | 0.673–0.832 |
| Physical | Q3 | Lift | 0.725 | 0.651–0.798 |
| Physical | Q4 | Bend forward | 0.749 | 0.671–0.826 |
| Physical | Q5 | Stand | 0.808 | 0.750–0.867 |
| Physical | Q6 | Sit | 0.692 | 0.612–0.772 |
| Physical | Q7 | Walk | 0.780 | 0.716–0.843 |
| Physical | Q8 | Run | 0.700 | 0.614–0.785 |
| Physical | Q9 | Sports | 0.819 | 0.760–0.877 |
| Physical | Q10 | Playing | 0.780 | 0.711–0.849 |
| Physical | Q11 | Ride a bike | 0.718 | 0.633–0.803 |
| Physical | Q12 | Not as much fun | 0.697 | 0.612–0.782 |
| Physical | Q21 | Energy | 0.695 | 0.613–0.778 |
| Psychosocial | Q13 | Lonely | 0.746 | 0.659–0.832 |
| Psychosocial | Q14 | Worried | 0.754 | 0.668–0.840 |
| Psychosocial | Q15 | Scared | 0.761 | 0.684–0.838 |
| Psychosocial | Q16 | Grumpy or annoyed | 0.738 | 0.659–0.818 |
| Psychosocial | Q17 | Sad or upset | 0.765 | 0.688–0.843 |
| Psychosocial | Q18 | Concentrate | 0.721 | 0.637–0.804 |
| Psychosocial | Q19 | School | 0.706 | 0.618–0.795 |
| Psychosocial | Q22 | Talk to mum and dad | 0.687 | 0.602–0.771 |
| Psychosocial | Q23 | Talk to friends | 0.727 | 0.650–0.803 |
| Psychosocial | Q24 | Treatment | 0.657 | 0.565–0.749 |

CFA, confirmatory factor analysis; CI, confidence interval. All 23 items were entered simultaneously in the prespecified correlated two-factor model using ordinal WLSMV estimation. All standardized factor loadings were statistically significant (p<0.001). No post hoc model respecification was performed.

# Supplementary Table S2. Item-level internal consistency statistics

| **Component** | **Item** | **Corrected item–total correlation** | **Cronbach’s α if item deleted** |
| --- | --- | --- | --- |
| Physical | Q1 | 0.712 | 0.923 |
| Physical | Q2 | 0.696 | 0.923 |
| Physical | Q3 | 0.683 | 0.924 |
| Physical | Q4 | 0.679 | 0.924 |
| Physical | Q5 | 0.742 | 0.922 |
| Physical | Q6 | 0.632 | 0.926 |
| Physical | Q7 | 0.727 | 0.922 |
| Physical | Q8 | 0.623 | 0.926 |
| Physical | Q9 | 0.749 | 0.922 |
| Physical | Q10 | 0.707 | 0.923 |
| Physical | Q11 | 0.657 | 0.925 |
| Physical | Q12 | 0.626 | 0.926 |
| Physical | Q21 | 0.627 | 0.926 |
| Psychosocial | Q13 | 0.646 | 0.892 |
| Psychosocial | Q14 | 0.679 | 0.890 |
| Psychosocial | Q15 | 0.683 | 0.889 |
| Psychosocial | Q16 | 0.695 | 0.889 |
| Psychosocial | Q17 | 0.654 | 0.891 |
| Psychosocial | Q18 | 0.624 | 0.893 |
| Psychosocial | Q19 | 0.612 | 0.894 |
| Psychosocial | Q22 | 0.637 | 0.892 |
| Psychosocial | Q23 | 0.691 | 0.889 |
| Psychosocial | Q24 | 0.607 | 0.894 |

Overall Cronbach’s α was 0.929 for the Physical component and 0.901 for the Psychosocial component. Deletion of any individual item did not increase the corresponding component-level Cronbach’s α.

# Supplementary Table S3. Prespecified construct-validity hypotheses and results

| **Hypothesis** | **Relationship** | **Prespecified expectation** | **Observed result** | **Confirmed** |
| --- | --- | --- | --- | --- |
| H1 | Physical component vs SRS-22 Function/Activity | \|ρ\| ≥0.50 | ρ=−0.769 | Yes |
| H2 | Physical component vs SRS-22 Pain | \|ρ\| ≥0.40 | ρ=−0.749 | Yes |
| H3 | Psychosocial component vs SRS-22 Mental Health | \|ρ\| ≥0.50 | ρ=−0.727 | Yes |
| H4 | Psychosocial component vs SRS-22 Self-image | \|ρ\| ≥0.30 | ρ=−0.634 | Yes |
| H5 | Physical vs Mental Health weaker than Psychosocial vs Mental Health | \|ρPhysical\| < \|ρPsychosocial\| | 0.510 < 0.727 | Yes |
| H6 | Psychosocial vs Function/Activity weaker than Physical vs Function/Activity | \|ρPsychosocial\| < \|ρPhysical\| | 0.524 < 0.769 | Yes |
| H7 | Physical and Psychosocial components vs Satisfaction with management | Both \|ρ\| <0.30 | ρ=−0.233; −0.245 | Yes |
| H8 | Sleep item vs each of five individual SRS-22 domains | 0.30 ≤ \|ρ\| ≤0.60 for every domain | Satisfaction: ρ=−0.225 | No |

Seven of eight prespecified hypotheses were confirmed (87.5%), exceeding the predefined 75% criterion for adequate construct validity. H8 was not confirmed because the Sleep–Satisfaction correlation was below the prespecified lower bound. Subtotal and total SRS-22 scores were treated as descriptive composite measures and were not used for formal hypothesis testing.

# Supplementary Table S4. Additional test–retest agreement and measurement-error analyses

**Panel A. Agreement between T1 and T2 for the Physical and Psychosocial components**

| **Measure** | **Mean difference (T1−T2)** | **95% limits of agreement** | **Wilcoxon p value** |
| --- | --- | --- | --- |
| Physical component | −2.97 | −17.93 to 12.00 | 0.003 |
| Psychosocial component | 0.76 | −17.82 to 19.35 | 0.738 |

**Panel B. Measurement-error estimates for Section 2 impact items**

| **Section 2 item** | **ICC(A,1) (95% CI)** | **SEM** | **MDC95** |
| --- | --- | --- | --- |
| Pain | 0.823 (0.718–0.891) | 0.653 | 1.809 |
| Physical activities | 0.864 (0.782–0.917) | 0.634 | 1.757 |
| Social relations | 0.813 (0.705–0.884) | 0.664 | 1.842 |
| Mood and concentration | 0.864 (0.783–0.917) | 0.624 | 1.731 |

ICC(A,1), two-way absolute-agreement single-measurement intraclass correlation coefficient; SEM, standard error of measurement; MDC95, minimal detectable change at the 95% confidence level. The mean difference was defined as T1 minus T2. SEM was calculated as baseline SD × √(1−ICC), and MDC95 as 1.96 × √2 × SEM.

# Supplementary Table S5. Exploratory sex measurement invariance and sensitivity CFA analyses

**Panel A. Exploratory sex measurement invariance**

| **Model** | **CFI** | **TLI** | **RMSEA (90% CI)** | **SRMR** | **ΔCFI** | **ΔRMSEA** |
| --- | --- | --- | --- | --- | --- | --- |
| Configural | 0.997 | 0.996 | 0.018 (0.000–0.039) | 0.062 | — | — |
| Thresholds | 0.996 | 0.996 | 0.019 (0.000–0.039) | 0.062 | −0.001 | +0.001 |
| Metric | 0.996 | 0.996 | 0.018 (0.000–0.038) | 0.062 | 0.000 | −0.002 |
| Scalar | 0.997 | 0.997 | 0.016 (0.000–0.037) | 0.062 | +0.001 | −0.002 |

**Panel B. Sensitivity CFA models**

| **Model** | **χ² (df), p** | **CFI** | **TLI** | **RMSEA (90% CI)** | **SRMR** |
| --- | --- | --- | --- | --- | --- |
| Two-factor correlated, 23 items | 243.37 (229), p=0.245 | 0.997 | 0.996 | 0.018 (0.000–0.035) | 0.045 |
| Physical only, one factor | 72.89 (65), p=0.235 | 0.997 | 0.997 | 0.025 (0.000–0.051) | 0.034 |
| Psychosocial only, one factor | 44.09 (35), p=0.139 | 0.995 | 0.993 | 0.036 (0.000–0.066) | 0.037 |

CFA, confirmatory factor analysis; CFI, comparative fit index; TLI, Tucker–Lewis index; RMSEA, root mean square error of approximation; SRMR, standardized root mean square residual. Sex invariance analyses were exploratory. Invariance was considered supported when ΔCFI was not less than −0.010 and ΔRMSEA was not greater than +0.015. The separate one-factor models were sensitivity analyses; the prespecified primary structural model was the simultaneous correlated two-factor CFA of all 23 Physical and Psychosocial items.

# Supplementary Figure S1A. Bland–Altman plot for the Physical component

The difference was defined as T1 minus T2. The mean difference was −2.97 points and the 95% limits of agreement were −17.93 to 12.00.

# Supplementary Figure S1B. Bland–Altman plot for the Psychosocial component

The difference was defined as T1 minus T2. The mean difference was 0.76 points and the 95% limits of agreement were −17.82 to 19.35.

# Supplementary Figure S2. Section 2 impact-item radar plot

The radar plot displays the median and interquartile range of the four Section 2 impact items in the full baseline sample (n=199). Section 2 items are interpreted individually and are not combined into a summary score. This figure is descriptive and does not establish clinical treatment tailoring.
